# Uncovering Enhancer Functions Using the α-Globin Locus

**DOI:** 10.1371/journal.pgen.1004668

**Published:** 2014-10-16

**Authors:** Douglas Vernimmen

**Affiliations:** The Roslin Institute, Developmental Biology Division, University of Edinburgh, Easter Bush, Midlothian, United Kingdom; University of Cologne, Germany

## Abstract

Over the last three decades, studies of the α- and β-globin genes clusters have led to elucidation of the general principles of mammalian gene regulation, such as RNA stability, termination of transcription, and, more importantly, the identification of remote regulatory elements. More recently, detailed studies of α-globin regulation, using both mouse and human loci, allowed the dissection of the sequential order in which transcription factors are recruited to the locus during lineage specification. These studies demonstrated the importance of the remote regulatory elements in the recruitment of RNA polymerase II (PolII) together with their role in the generation of intrachromosomal loops within the locus and the removal of polycomb complexes during differentiation. The multiple roles attributed to remote regulatory elements that have emerged from these studies will be discussed.

## Introduction

Developmentally regulated genes become activated only in the appropriate lineage, while they remain inactivated (or become fully repressed) in other lineages. Haematopoiesis offers an excellent system to study gene regulation because it is very accessible and produces a palette of a least eight different lineages in the blood [1]. Also, differentiated cells and their progenitors can be easily separated and purified using different cell surface markers [2], [3]. The erythroid cell lineage is of particular interest because of the high expression level of the globin genes, which produce the major proteins found in red blood cells. Adult haemoglobin (HbA) is made by the formation of a tetramer containing two α-globin chains and two β-globin chains. The level of expression of these proteins needs to be equimolar to ensure the correct formation of this tetramer; an imbalance would create insoluble homotetramers, the key pathological feature of thalassaemia [4].

The expression of adult α- and β-globin genes requires a panel of different tissue-specific transcription factors (TFs), including GATA1, GATA2, nuclear factor-erythroid 2 (NF-E2), stem cell leukaemia factor (SCL), and Krüppel-like factor 1 (KLF1) (formerly called Erythroid Krüppel-like factor [EKLF]). These TFs are expressed at different times during differentiation, suggesting specific roles for each [5]. For example, GATA2 is expressed early, in common myeloid progenitors, whereas GATA1 is expressed later, in erythroid progenitors. KLF1 is also expressed late during erythroid differentiation and therefore is important for late events in erythropoiesis such as the expression of the α- and β-globin genes [6], [7].

Enhancers were originally defined as sequences that increase the rate of transcription of a target gene [8]–[10]. They may lie far away upstream or downstream from the gene they regulate [10] and should work in both orientations [11]. These original definitions were based on reporter assays (i.e., plasmids) in which the distance separating enhancers and promoters is very small and the chromatin context is not taken into account. The activity in both orientations was primarily due to the fact that simple enhancers were identified as regions formed by a palindromic sequence (e.g., binding site of a homodimer [12]). It became more obvious later that enhancers usually work in groups (i.e., locus control region (LCR) and super enhancers [13]), each being bound by several TFs, forming a so-called enhanceosome [14], [15]. These enhanceosomes are nucleated by pioneer TFs early during differentiation and subsequently are replaced by other TFs that trigger transcription by PolII recruitment. More recently, in vivo studies found that enhancers (remote regulatory elements) can be located sometimes up to 1 Mb away from the gene they regulate [16]–[18]. What do these sequences do? How do they function across very large distances? These have been questions of major interest over the last two decades. Studies on the α- or β-globin loci have been pioneers in this field, and many approaches and tools have been developed to address this.

Overall, three main strategies have been used to understand the molecular mechanisms involved in the activation of the globin genes. The first involves biochemistry and molecular biology using different cell types representing different stages of differentiation and the analysis of TF complexes, TF binding patterns, chromosome conformation, and epigenetic changes. The second involves mouse genetics, and the third strategy involves the study of patients carrying different types of mutations (in these cases deletions) associated with the downregulation of expression of globin genes leading to thalassaemia. This review will be focused on studies made on both the mouse and the human α-globin loci and more particularly on the role attributed to the remote regulatory elements controlling their expression.

## Structure of the α-Globin Locus

**Figure 1:**
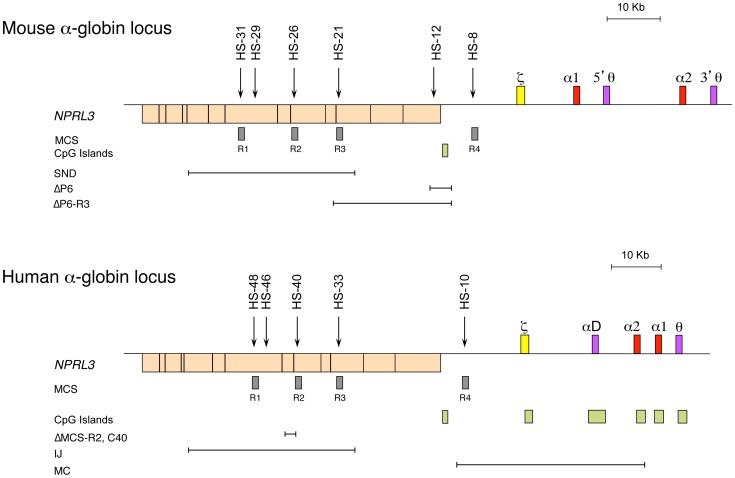
The chromosomal organisation of the mouse (top) and human (bottom) α-globin clusters. The embryonic ζ gene in each locus is represented in yellow, the pseudogenes in purple, and the foetal/adult α genes in red boxes. The positions of DNase I hypersensitive sites, discussed in the text, are shown as arrows. The widely expressed gene *NPRL3* is transcribed from the opposite strand to that of α-globin and is shown as an orange box, with exons in black bars. Grey boxes refer to previously defined multispecies conserved elements (MCS), and light-green boxes indicate CpG islands. Note the lack of CpG islands in the mouse locus. The previously described natural (IJ, MC) and targeted (C40, ΔMCS-R2) deletions from the human and targeted deletions from the mouse (ΔP6, ΔP6-R3, and SND) α-globin cluster are shown as annotated.

Studies of both the mouse and the human α-globin loci offer complementary advantages. The mouse locus can be manipulated easily, and the repertoire of cell lines is greater, whereas the human locus can be studied in samples obtained from patients [4]. The α-globin locus of all mammalian species analysed lies within a region of 135–155 kb of conserved synteny, with the α-like genes arranged along the chromosome in the order 5′-ζ-α-α-3′ (Figure 1) [19]. However, the mouse and human loci also show some important structural differences. The mouse locus has an arrangement containing two pseudogenes θ (5′-ζ-α1-5′θ-α2-3′θ-3′, Figure 1), whereas the human has an arrangement containing only one pseudogene θ, with another pseudogene (αD) that precedes the adult α genes (5′-ζ-αD-α2-α1-θ-3′). The αD gene is expressed in erythroid cells [20]. Also, the human α-like genes are each covered by a CpG island (Figure 1), which has a strong influence on their regulation (see below). The erythroid-specific multispecies conserved sequences (MCS) identified by DNase hypersensitive sites (DHS) have been numbered MCS-R1 to MCS-R4 [19] (Figure 1). Three of these elements (MCS-R1, MCS-R2, and MCS-R3) lie within the body of a housekeeping gene, *NPRL3*, and MCS-R4 lies upstream of the promoter of that gene (Figure 1). However, the mouse locus contains an additional DHS, located 12 kb upstream of the ζ gene (HS –12), that appears to be important during the priming of the locus during differentiation [5].

The globin gene disorders (haemoglobinopathies), including thalassaemias, are among the most common human genetic diseases, with more than 300,000 severely affected individuals born throughout the world every year [21]. Thalassaemias are characterized by inherited mutations leading to a reduction of the synthesis of α- (α-thalassaemia) or β-globin (β-thalassaemia) chains from one allele. Human genetics is a good approach to identify functional remote regulatory elements, and original observations were made in patients with α- and β-thalassaemia [22]–[24]. In most cases, a deletion removing a globin gene is the cause of this down-regulation, but in some rare cases, the genes (including their promoters) remain intact [25].

In rare cases of α-thalassaemia, further chromosome mapping in a number of patients led to the identification of other deletions located far upstream, overlapping the *NPRL3* housekeeping gene [26] and therefore the remote erythroid hypersensitive sites (Figure 1). By comparing all the different deletions in the *NPRL3* gene, it appears that MCS-R1 and MCS-R2 are consistently removed in all cases, and this led to the characterisation of these sequences [27], [28]. From these observations, different approaches, such as transient transfections and transgenics, have showed that MCS-R2 (previously called HS –40) has a much stronger enhancer activity. Moreover, this element shows a remarkable conservation throughout evolution. In zebrafish, it already lies in the intron of the *NPRL3* gene and drives the expression of both α- and β-globin on the same locus through a bidirectional promoter, securing comparable levels of α- and β-like globin protein [29]. The globin locus in fish has then diverged over time and segregated into separate α and β loci after the divergence of amphibians 450 million years ago, leaving the ancestral globin locus as an α-globin locus [30], [31]. Therefore, further efforts were concentrated on the characterisation of the ancestral MCS-R2 element in detail [28], [32]–[34].

## Epigenetic Control of α-globin Gene Expression

The epigenetic programme seems to play a key role in determining cell fate, including the decision to undergo self-renewal or commitment. Chromatin immunoprecipitation (ChIP) followed by high-throughput sequencing (ChIP-Seq) studies have suggested that the chromatin associated with many genes controlling lineage fate decisions is uniquely marked in stem cells [35]. Their histone signature includes modifications associated with both transcriptional repression (H3K27me3) imposed by the polycomb group proteins (PcG) and activation (H3K4me3) mediated by the trithorax group proteins (TrxG) [36]. The “permissive” chromatin state of these genes in stem cells is called bivalent and is supposed to be resolved during differentiation, as genes become fully activated or repressed [37]–[39]. The recruitment of complexes such as PcG and TrxG involves the presence of a CpG island at the target promoter of a developmentally regulated gene, and proteins with a CXXC domain binding to the DNA [40]–[44]. The maintenance and propagation of an epigenetic mark, such as H3K27me3 by polycomb repressive complex 2 (PRC2), is well documented and involves a “reader” protein (EDD), which recognises a modified histone, and a “writer” protein (histone methyltransferase Ezh2), which modifies the histones nearby [45]. The removal of such a mark by an “eraser” protein (histone demethylases JMJD3 and UTX) would prevent the maintenance and propagation from occurring. The human α-globin genes are associated with a CpG island, whereas in rodents (e.g., mouse and rat) this CpG island has been lost during evolution (Figure 1) [43]. Therefore, the presence of a CpG island on the human locus has an important implication in the epigenetic regulation of that locus (see below).

In mouse embryonic stem (ES) cells, the analysis of the mouse α-globin locus initially suggested it to be unmarked by any histone modifications such as H4ac or H3K4me2 [5]. More recent genome-wide studies (ChIP Seq) have shown that in ES cells, remote regulatory elements of a small number of developmentally regulated genes are already marked by H3K4me1, and in the mouse α-globin locus, a deposition of H3K4me1 has been found just next to MCS-R1 (HS –31) in ES cells [46], suggesting a possible priming around that region.

In human ES cells and in nonerythroid cells, the CpG island is associated with PRC2 recruitment and its associated signature (H3K27me3), whereas these are not found in the rodent α-globin genes [47]. In human ES cells, the α-globin CpG islands are also covered by H3K4me3. However, the domains of H3K27me3 and H3K4me3 do not entirely overlap, and the ratio observed progressively changes during differentiation [48], suggesting that the progressive increase of H3K4me3 could be due to an expression of the gene at irrelevant stages rather than a signature of priming, as suggested by previous studies [36], [49]. Therefore, single-cell mRNA expression in human ES cells was measured and revealed that although full length α-globin mRNA was detected in a small number of Oct4 positive cells, this number increased during differentiation. This suggested that some bivalent domains might be the consequence of a subpopulation phenomenon rather than true priming in all cells [48]. PcG complexes remain until the last stages of differentiation [48].

## Locus Priming during Differentiation

**Figure 2:**
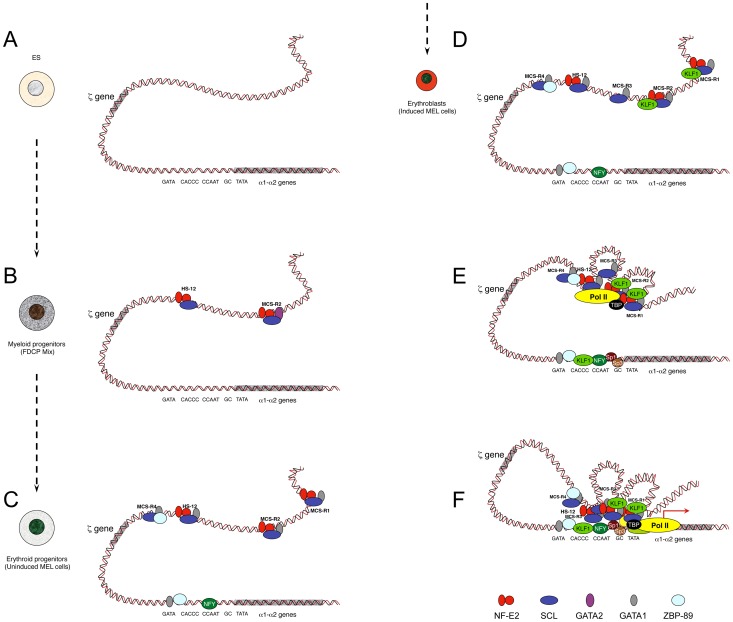
Model showing sequential order of TF binding on the mouse α-globin locus during erythropoiesis. The locus has been analysed in pluripotent cells (A), myeloid progenitors (B), committed erythroid progenitors (C), and in differentiating erythroid cells (D, E, F). Note that D, E, and F do not represent different steps during activation but simply separate illustrations representing the formation of MCS-R3 and recruitment of KLF1 (D), the recruitment of PolII at the enhancers independently of the promoter (E), and a multilooped structure involving enhancers and promoters (F). Note that the human α-globin locus has been mainly characterised in human ES cells and primary erythroid cells. It is still not known if MCS-R2 is also primed in human progenitor cells (i.e., myeloid progenitors) as it is for the mouse locus. Studies have shown that the TF binding pattern is essentially the same with two main key differences: GATA1 does not bind the human promoter in erythroid cells, and the gene is repressed by PcG complexes in human ES cells. PcG complexes are removed late during erythropoiesis. For the sake of clarity, the locus has been represented as a double helix of DNA rather than by a 10 nm chromatin fibre.

Developmentally regulated genes are progressively primed during the differentiation programme, a process that involves the sequential recruitment of stage-specific TFs associated with histone modifications. The study of α-globin was initiated using the mouse locus and a panel of different cell lines and primary cells that represent the different stages of erythroid differentiation (Figure 2) [5]. As a source of multipotent cells, mouse ES cells have been used, and so far no TFs have been found bound within the mouse α-globin MCS-Rs. This contrasts with other single-locus studies such as the mouse β-globin locus [50] and the λ5-VpreB1 locus [51] showing priming at enhancers (including PolII recruitment) in ES cells, long before transcription occurs (differentiated erythroid and B cells, respectively).

As a source of common myeloid progenitor cells (CMP), factor dependent cell Patterson (FDCP)–mix cells have been used [52]. These are able to produce both megakaryocytic and erythroid lineages. In these cells, the mouse α-globin locus is primed at MCS-R2 and HS –12 by GATA2 (Figure 2B) [5]. During differentiation, GATA2 regulates the expression of GATA1 with a negative feedback loop, which shuts down GATA2 expression in proerythroblasts [53]. As a source of proerythroblasts, the MEL (mouse erythroleukemia) cell line has been used. This cell line was originally transformed by the Friend virus [54] and is a very useful model to study the last stages of erythroid differentiation since, like most blood cell lines, it can be induced towards terminal differentiation after exposure to certain chemicals. Under treatment with inducing agents, such as dimethyl sulphoxide (DMSO) or hexa-methylene bis-acetamide (HMBA), MEL cells turn a red colour because of haemoglobinisation. At the proerythroblast stage (uninduced MEL), most remote regulatory elements are formed (HS –12, MCS-R1, MCS-R2, and MCS-R4) with the recruitment of key erythroid TFs, including GATA1, NF-E2 (dimer p45 and p18), and the SCL pentameric complex (Figure 2C) [5]. The promoter also becomes accessible and bound by GATA1, 89-kDa zinc finger protein (ZBP-89), and nuclear factor Y (NFY) (CAAT-box-binding protein). The human α-globin promoter does not contain an evolutionarily conserved GATA site and does not bind ZBP-89, but NFY binding is common in both species [7]. NFY has a histone-like structure [55] and therefore could be involved in chromatin opening by nucleosome replacement [56]. In erythroblasts (induced MEL), more changes occur, with the appearance of MCS-R3 (HS –21), together with recruitment of KLF1 (Figure 2D) and the preinitiation complex (PIC) at both enhancers and promoters in the human and the mouse α-globin loci (Figure 2E) [7]. At that final stage, other Sp/X-Kruppel-like transcription factors (Sp/X-KLFs) are also recruited to the α-globin promoters [57], [7]. Interestingly, a looped structure that bridges the remote regulatory elements to the promoter was also detected by chromosome conformation capture analysis using quantitative Taqman technology (q3C) at that particular stage of differentiation (Figure 2F) [7], where high level of α-globin expression occurs. *NPRL3* gene expression is also up-regulated in human [58] and mouse [59] erythroid cells and associated with bidirectional intergenic transcripts at these elements (enhancer RNA [eRNA]) [59]. The more recent advances of ChIP-Seq technologies allowed other groups to find the same phenomenon with peaks of PolII in intergenic regions [60]–[62] associated with H3K4me1, a key feature of enhancers [63]. Note that the characterization of the human α-globin locus in progenitor cells has not been characterized to the same depth as in differentiated cells because the repertoire of progenitor cells is less accessible. Indeed, progenitor cells can be obtained from bone marrow samples from individuals undergoing total hip replacement for osteoarthritis [48] or after mobilisation for bone marrow transplant, but the amount of cells collected is still very limited.

## Role(s) of the Remote Regulatory Sequences

The natural deletions observed in patients were an essential tool in the analysis of the role of these remote regulatory elements. Due to the nature of the disease (anaemia), it was however difficult to perform a number of experiments on primary cells because of the large amount of material required and also because this material is only available for a limited time. For this reason, interspecific hybrids were produced by the fusion of a human immortalised B cell (Epstein-Barr virus [EBV] infected) with the mouse MEL cell line described earlier [24], [28], [64], [65].

Therefore, interspecific hybrids, derived from normal individuals or those with previously characterised natural mutations of the α-globin cluster, were analysed to determine if the PIC was recruited independently both at the promoter and at the remote regulatory elements. Two types of mutation were analysed: one in which the remote upstream elements had been fully (IJ) deleted but the α promoters remained intact [27], [66] and another in which all α-like genes were deleted (MC) but the upstream elements were still present (Figure 1). From these studies, two important principles in the hierarchy governing the process of transcription activation have been uncovered. First, the presence of the remote enhancers is required for the recruitment of the PolII at the promoter. Secondly, the presence of the promoter is not required for PolII recruitment at the enhancers [7]. In other words, the recruitment of PolII at the enhancers occurs independently of the promoter. In a similar way, preventing PolII recruitment at the mouse β-globin promoter does not affect its binding at the upstream LCR [67]. This important feature was validated later on a study on the *Arc* locus [61] where, in the *Arc* gene knockout neurons, PolII remains bound at the Arc enhancer at levels equivalent to those observed in wild-type neurons. In the former study, eRNA synthesis is abolished in the absence of the gene, suggesting that, like mRNA synthesis, eRNA synthesis may require an interaction of the enhancer with a promoter [61]. However, another study on the human growth hormone (*hGH-N*) locus showed that this is not always the case [68].

The use of interspecific hybrids is a useful way to study enhancer functions; however, they are very difficult to maintain, mainly because they tend to lose the human chromosome 16 and they are still also very difficult to induce into erythroblasts. More recently, another mouse model was generated in which the whole region of conserved synteny (87 kb of mouse genomic DNA) was replaced by the human orthologous region (117 kb of human genomic DNA) [69]. Therefore, a humanized mouse was produced, which offers the advantage to avoid the issues generated by the use of conventional transgenics [69], [70]. Although the spatiotemporal expression of the human α-globin was correct, the level seemed to be suboptimal at about 50% of what was expected. However, in this model, the pattern of TF binding (including PolII) and the looped structure seem to be identical to that in human primary erythroblasts [71], [72]. This new model was therefore the best one to use so far to investigate the role of MCS-R2. A deletion of about 1.1 kb covering MCS-R2 was made in heterozygous mice (one humanised chromosome and one normal mouse chromosome). In this situation, the level of α-globin expression reached only ∼2% compared to the normal humanised chromosome [69], [71]. These data recapitulated well the previous data obtained with other models, including a hybrid clone bearing the same deletion (Figure 1, C40 hybrid [27]).

The changes of TF binding and chromosomal looping were then analysed in normal humanised mice and compared to humanized mice without MCS-R2 (ΔMCS-R2) [73]. In the ΔMCS-R2 mutant, the absence of α-globin expression is associated with a lack of PolII binding together with an impaired looping formation. The occupancy of PolII from the start to the end of the gene is about 6%–3% of normal, matching the amount of mRNA expressed, and therefore ruled out any role of MCS-R2 in PolII elongation. A role in PolII elongation was originally suggested for the LCR of the β-globin locus [74]. In this study, a targeted deletion of the human LCR in transgenic mice reduces PIC recruitment of about 50% to normal but with a more dramatic effect on Ser5 phosphorylation of PolII (Ser-5P) and transcriptional elongation, suggesting a role for the β-globin LCR in both PolII recruitment (partially) and elongation. Similar observations were made later with the mouse β-globin locus, in which a targeted deletion of the mouse LCR reduces global PolII recruitment at the promoter to 30% of normal with a redistribution of Ser-2P along the gene [75]. Recently, the Furlong group showed that in *Drosophila* enhancers would loop towards a target promoter to recruit PolII but paused in the majority of cases. Releasing of PolII pausing would then occur by subsequent recruitment of additional TFs or additional enhancers [76]. Note that in the studies of the α-globin locus, kinases regulating PolII such as cdk7 (TFIIH), cdk-8 (mediator), and cdk9 (elongation factor p-TEFb) are all recruited at the same time on the promoter when looping and transcription occur [7]. On the mouse β-globin locus, cdk9 and PolII Ser-2P binding is observed at both the LCR and the promoter when the gene is active [75]. Although the binding of cdk9 to the LCR precedes its binding to the promoter, the deletion of the LCR does not affect cdk9 binding at the promoter. This study would suggest that the β-globin LCR may not control elongation by delivery of cdk9 but would involve other complexes such as DRB sensitivity inducing factor (DSIF) and facilitates chromatin transcription (FACT) [77]. Another study on the Myb locus in erythroid cells [78] together with a genome-wide study in human B lymphoblast cells (MM1.S) [79] suggested an important role of cdk9 bound to enhancers. Other genome-wide studies detected one (Ser-5P) [60] or both (Ser-2P and Ser-5P) [80] phosphorylated forms of PolII at active enhancers, supporting the idea that enhancers would deliver an activated PolII at the target promoters. A general role of remote enhancers in PolII delivery to a target promoter has been also described in other loci such as the human growth hormone (*hGH*) locus [81], [82], the human serpin cluster [83], and the mouse T cell receptor beta (TCRβ) locus [84].

**Figure 3:**
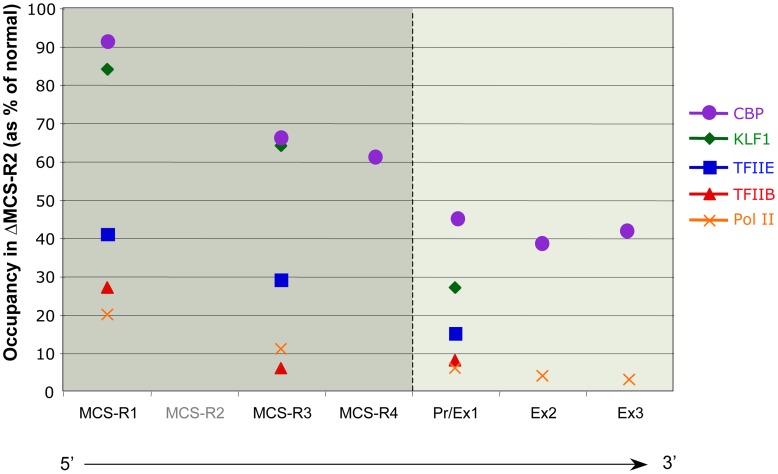
Differential occupancy of TFs in the absence of the enhancer (MCS-R2) in mouse primary erythroblasts. The percentages of occupancy in the mutant human allele (ΔMCS-R2) was calculated by comparison with the normal human allele ( = 100%) after normalising the ChIP efficiency to an endogenous control (mouse α-globin allele). This graph shows a reduction of TFs occupancy (5′→3′) with the distance along the locus, with the lowest occupancy observed at the gene (Pr/Ex1 – Ex2 – Ex3). Figure adapted from Vernimmen et al., 2011; Genes and Development [73].

**Figure 4:**
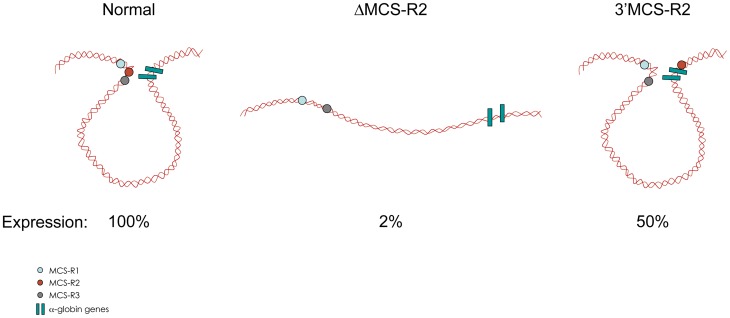
Communications between enhancers influences transcription. Intrachromosomal interactions involving human remote regulatory sequences and the human α-globin genes in normal, ΔMCS-R2, and 3′MCS-R2 humanised alleles in mouse primary erythroblasts.

In the ΔMCS-R2 mutant, the PolII occupancy at the other remaining enhancers is also affected but not as much as at the promoter. In fact, the MCS-R1 element, located upstream of the deletion, is less affected (∼20% of normal PolII occupancy) than the other regions downstream (MCS-R3, MCS-R4, and the promoter). The same trend is observed with other components of the PIC and other TFs such as KLF1 (Figure 3) [73]. Strikingly, this same observation was previously made with the C40 hybrid, albeit with a less dramatic reduction of TF binding (general transcription factors [GTFs] are unchanged at MCS-R1, and PolII occupancy level at the promoter is of about 20% compared to normal; [7], see discussion below). This reduction of TF occupancy across the locus suggests that the activating signal is propagating from the upstream elements towards the downstream promoter. The nature of this signal polarity is not yet understood and still has to be tested experimentally. Note, a polarity has been shown with the β-globin LCR locus, which works in an orientation-dependent manner [85]. Although this could also suggest a form of facilitated tracking, involving a unidirectional (multi)looped structure progressing towards the downstream promoters [86]–[89], there was no evidence of TF binding in the intervening DNA regions. Keeping this idea in mind, MCS-R2 was reinserted in an ectopic site in the region of conserved synteny, just downstream from the α-globin genes (Figure 4) [71]. In this new mutant (3′ MCS-R2), transcription is reactivated to about ∼50%, and more importantly, the looped structure is reestablished. This would argue against a facilitated-tracking model since, in this situation, the other remote elements (MCS-R1, MCS-R3, and MCS-R4) interact again with MCS-R2 and the α-globin genes, both located on the other side of the loop (Figure 4).

The analysis of MCS-R2 was completed by investigating its potential role in the deposition and/or removal of histone modifications, which are associated with activation and repression. Since the literature has reported about 150 possible histone modifications [90], this α-globin study concentrated on the main modifications associated with activation (H3 and H4 acetylation, H2B ubiquitination, H3K79me3, H3K4me3, and H3K36me3) and repression (H3K27me3). Repression associated with polycomb repressive complex 1 (PRC1) was not investigated because of the poor efficiency of the antibodies against PRC1 proteins and their associated modification (H2Aub). In agreement with the very low expression of the gene without MCS-R2, modifications such as H2Bub and H3K79me3 do not occur. Histone 3 mono- and di-methyl K79 are less affected by the deletion and are also found at the other remote regulatory sequences, whereas H3K79me3 was exclusively found at the body of the gene. Surprisingly, all modifications generated by the TrxG pathway (H3K4me1, H3K4me2 and H3K4me3) occur normally in the absence of MCS-R2. Histone 3 tri-methyl K4 is, however, suboptimal at the body of the gene, and H3K4me1 is not only detected at the other MCS-R but is also significantly enriched at the body of the gene. It is worth noting that these are both common features observed in ES cells [48].

**Figure 5:**
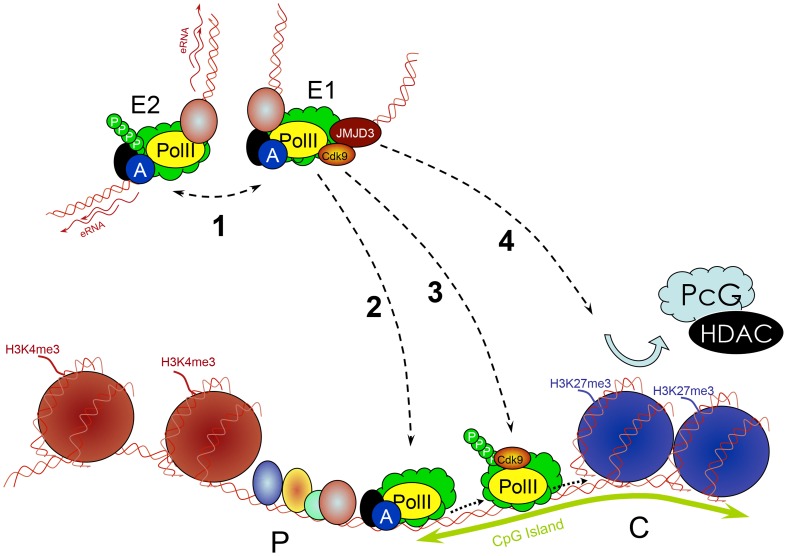
Multiple roles of enhancers. Enhancers (E) work in groups. They recruit PIC and TFs and interact with each other (1), which is associated with increased PolII concentration and production of eRNA. Enhancers subsequently deliver the PIC to a target promoter (P) through a looped structure (2). They control transcription elongation of some genes (e.g., by delivering an important kinase such as cdk9) by increasing the phosphorylation of PolII (3). They remove PcG repressive complexes from CpG islands of developmentally regulated genes through a histone demethylase (e.g., JMJD3) removing H3K27me3 (4).

It was originally thought that the generation of H3K4me3 in ΔMCS-R2 mutant is due to the presence of the CpG island, since it was previously reported that the α-globin genes were already covered by this activating mark in human ES cells [47]. Moreover, the work from Adrian Bird's group has shown that a CXXC domain protein, cfp1 (also called CpG binding protein [CGBP]) is involved in the deposition of H3K4me3 at the CpG island of target genes regardless of their transcription status [44]. CGBP is part of a TrxG complex (human Set1) and therefore would generate H3K4me3 at any CpG island. In ChIP analysis, CGBP is only detected at the α-globin CpG island in the presence of MCS-R2 [73], suggesting another mechanism for H3K4me3 deposition in ΔMCS-R2 mutant. It was also reported that the binding of CGBP could be mutually exclusive with other proteins binding to CpG islands such as PcG [44]. Indeed, in the absence of MCS-R2, PRC2 (including histone deacetylase 1 [HDAC1]) recruitment continues at the α-globin CpG island throughout the whole process of erythroid differentiation, whereas this complex is removed in the presence of MCS-R2 enhancer (Figure 5). Importantly, the removal of PRC2 and H3K27me3 was associated with the recruitment of the H3K27me3 demethylase JMJD3 at the CpG island [73].

## Conclusions

Using α-globin as a model, the study of enhancer biology was made possible by using cell lines and primary cells that faithfully represent the different stages of erythropoiesis. The sequential order of TF binding, with the appearance of DHSs, is followed by the recruitment of PolII at both enhancers and promoter at the last stages of erythropoiesis, when globin expression occurs. This contrasts with studies on the mouse β-globin and the λ5-VpreB1 loci in which PolII is also recruited late at the promoter but is detected very early at the remote regulatory elements [50], [51]. Using human genetics, it has been possible to show that the recruitment of PolII at the remote enhancers occurs independently of the promoter. The general idea that enhancers act as docking sites for the recruitment of the general transcription machinery (components of the PIC and not just PolII) has been suggested in a few studies [60], [91], [92].

Once the PIC is recruited to the enhancer, this transcription machinery would be then somehow transferred to the downstream promoter by a looping mechanism. Note that although the duplicated α-globin genes in most species have similar or identical promoters, the gene closest to the upstream elements is usually expressed at the higher level (reviewed in [93]). When more than two genes are present, the additional genes lying downstream appear to be expressed at even further decreased levels [94] and thus suggest the possibility of a local tracking process involved. However, this would be unlikely since the terminator element of the first gene should block this tracking [81], and the second gene (in the mouse) is flanked by CTCF [95], which should also block such a tracking mechanism [87]. By using both samples from patients and humanised mice bearing a deletion covering the major remote element (MCS-R2), it has been possible to show that the enhancer is required for the recruitment of the PIC and key TFs at the promoter. How the PIC is recruited to enhancers is still not known. An in vitro study on the β-globin locus suggested that TFs bound to enhancers are able to recruit directly the PIC [96], but in vivo studies on the same locus suggest that the β-globin LCR facilitates the localisation of the locus in active transcription sites termed transcription factories [77]. Both the α- and β-globin loci are associated with active transcription sites in the nucleus of living cells [97], but it is still left to debate if these transcription factories are simply reflecting focal accumulations of PolII on transcribing genes or preassembled organising structures to which genes move (reviewed in [98]).

Developmentally regulated genes are controlled by several enhancers, recently termed super enhancers [99], and q3C analysis showed that α-globin enhancers also communicate with each other (Figure 2E and Figure 4) [71]. These enhancers are primed at different times during differentiation, and this might create a sort of directionality of the signal. Interestingly, the deletion of MCS-R2 creates a reduction of TF binding downstream, across the locus (Figure 3) [73]. This could be the result of an impaired multilooped structure involving all the enhancers and the two α-globin promoters together. What ties the loop on the α-globin locus still has to be determined. Many proteins involved in the interactions between enhancers and promoters have been described (reviewed in [100]), and the role of each on the α-globin locus still has to be investigated.

CpG islands were originally found in housekeeping genes and are involved in keeping the chromatin open and therefore allowing basic transcription to occur [101]. However, we now know that CpG islands are also found in the promoters of developmentally regulated genes, which are not always active. For this group of CpG island promoters, PcG act to block the promoter accessibility in inappropriate lineages or at other stages during the differentiation programme. Nevertheless, it is not clear why developmentally regulated genes should have CpG islands in the first instance. In stem cells, the level of H3K4me3 observed at the human α-globin gene is indeed associated with full-length transcripts, albeit at a very low level [48]. The mouse α globin seems to be perfectly regulated in the absence of a CpG island. Although the level of full-length transcripts increases together with H3K4me3 during differentiation, H3K27me3 and PcG complexes seem to remain until the last stage of differentiation. The humanised mouse model made it possible to show that the removal of this repressive mark was dependent on the MCS-R2 enhancer, together with the recruitment of demetylase JMJD3 [73]. Of interest, another mutant with MCS-R2 deletion (C40 hybrid) did not show any persistence of PcG at the α-globin gene (unpublished data). This deletion, however, was made in a hybrid cell line [27], corresponding to the proerythroblast stage, which was described earlier (Figure 1). Thus, depending on the timing when the deletion was made (ES cells, i.e., prior to development versus proerythroblats, i.e., after differentiation), different results can be found [102]. Interestingly, the level of transcription in the absence of MCS-R2 was similar, and therefore the increased level of transcription might not be the cause of PcG eviction. Also, the fact that PcG eviction is associated with the recruitment of a H3K27me3 demethylase suggests that it is more likely to be an active process [73]. It is therefore possible that enhancers could also recruit enzymes that remove histone modifications generated by PcG complexes at the target promoter after chromosomal looping, as it was suggested for the PIC. JMJD3 enrichment is not detected at MCS-R2 in humanized mouse erythroid cells by ChIP-qPCR, although this is at a much lower resolution [73]. However, ChIP-Seq studies showed enrichment of JMJD3 at both genes and distal intergenic regions [103], [104]. The role of remote enhancers in the eviction of repressive PRC2 and PRC1 is also supported by other recent studies [105]–[107].

This all together led to the conclusion that MCS-R2 has multiple roles, and these may be applicable to any other enhancer: recruitment of PolII and key TFs at the promoter, formation of a looped structure involving several remote regulatory elements, and the removal of repressive complexes such as PcG (Figure 5). It thus seems that enhancers have evolved with the overall complexity of mammalian transcription regulation by developing multiple roles required for optimal gene expression. These different roles might be allocated to different enhancers controlling the same gene; some might be important for initiation of gene expression, others for its maintenance. How to disentangle these roles remains a challenge, since most of the time many events occur at the same time during differentiation. Alternative models allowing short kinetics (e.g., lipopolysaccharide (LPS)-activated macrophages [108]) might be more suitable for the study of gene activation when applicable. Indeed, the latest reflects gene activation in a given cell type that is under a physiologic change, whereas globin genes activation occurs with a change of cell identity during differentiation.

## Future Directions

Over the last five years, high-throughput sequencing technology has been used to characterise human genetic diseases using materials from patients but also has provided a very broad picture of the chromatin landscape in many cell types. However, functional analyses are still required to provide mechanistic insights about enhancer functions. Over the last 30 years, single-locus dissection such as what has been achieved at the α- and β-globin loci remains a good example of where human genetics and genetic engineering in the mouse can provide important answers on how mammalian genes are regulated during differentiation and development. This is also applicable to other loci. For example, studies on patients with preaxial polydactyly led to the identification of the regulatory element controlling the expression of sonic hedgehog (SHH) in the posterior part of the limb [109]. Identifying individuals with α-thalassaemia by regular screenings allowed the mapping of all the known mutations, but sometimes new rare variants can be found, and these can provide new mechanisms underlying human genetic disease [21]. Human genetics, although powerful, still adds a layer of complexity when genetic variability between individuals has to be taken into account. Recently, a few rare cases of patients homozygous for MCS-R2 deletion have been described [110], [111]. Surprisingly, these patients present a phenotype less severe than expected, even with a broader deletion removing the other remote regulatory elements on one chromosome [110]. This demonstrates that the other regulatory elements must have a role but also suggests that, when deleted, “orphan” enhancers might relay for α-globin expression as recently suggested for other genes (enhancer adoption [112]). It is worth noting that in humans another enhancer-like element has been found outside the region of conserved synteny, 400 kb downstream from the α-globin locus [58]. This element lies inside the intron of another host gene called NME4, which is up-regulated in erythroid cells through an alternative start site, as is the case for the upstream *NPRL3* gene [58], [113]. Would this element take an important role when the others are missing? This is a difficult question to address, since this gene lies on another chromosome in the mouse and therefore no suitable model to test this hypothesis is currently available.
